# A Hybrid Intrusion Detection Model Using EGA-PSO and Improved Random Forest Method

**DOI:** 10.3390/s22165986

**Published:** 2022-08-10

**Authors:** Amit Kumar Balyan, Sachin Ahuja, Umesh Kumar Lilhore, Sanjeev Kumar Sharma, Poongodi Manoharan, Abeer D. Algarni, Hela Elmannai, Kaamran Raahemifar

**Affiliations:** 1 Chitkara University Institute of Engineering and Technology, Chitkara University, Punjab, India; 2 KIET Group of Institutions, Delhi-NCR, Ghaziabad 201206, India; 3 Division of Information and Computing Technology, College of Science and Engineering, Hamad Bin Khalifa University, Doha 500001, Qatar; 4 Department of Information Technology, College of Computer and Information Sciences, Princess Nourah bint Abdulrahman University, P.O. Box 84428, Riyadh 11671, Saudi Arabia; 5 College of Information Sciences and Technology, Data Science and Artificial Intelligence Program, Penn State University, State College, PA 16801, USA; 6 School of Optometry and Vision Science, Faculty of Science, University of Waterloo, 200 University Ave W, Waterloo, ON N2L3G1, Canada; 7 Faculty of Engineering, University of Waterloo, 200 University Ave W, Waterloo, ON N2L3G1, Canada

**Keywords:** Hybrid IDS, genetic algorithm, particle swarm optimization, random forest, machine learning, intrusion detection, security

## Abstract

Due to the rapid growth in IT technology, digital data have increased availability, creating novel security threats that need immediate attention. An intrusion detection system (IDS) is the most promising solution for preventing malicious intrusions and tracing suspicious network behavioral patterns. Machine learning (ML) methods are widely used in IDS. Due to a limited training dataset, an ML-based IDS generates a higher false detection ratio and encounters data imbalance issues. To deal with the data-imbalance issue, this research develops an efficient hybrid network-based IDS model (HNIDS), which is utilized using the enhanced genetic algorithm and particle swarm optimization(EGA-PSO) and improved random forest (IRF) methods. In the initial phase, the proposed HNIDS utilizes hybrid EGA-PSO methods to enhance the minor data samples and thus produce a balanced data set to learn the sample attributes of small samples more accurately. In the proposed HNIDS, a PSO method improves the vector. GA is enhanced by adding a multi-objective function, which selects the best features and achieves improved fitness outcomes to explore the essential features and helps minimize dimensions, enhance the true positive rate (TPR), and lower the false positive rate (FPR). In the next phase, an IRF eliminates the less significant attributes, incorporates a list of decision trees across each iterative process, supervises the classifier’s performance, and prevents overfitting issues. The performance of the proposed method and existing ML methods are tested using the benchmark datasets NSL-KDD. The experimental findings demonstrated that the proposed HNIDS method achieves an accuracy of 98.979% on BCC and 88.149% on MCC for the NSL-KDD dataset, which is far better than the other ML methods i.e., SVM, RF, LR, NB, LDA, and CART.

## 1. Introduction

For decades, ID has been studied in computer security research. The latest development of ML has enhanced the efficiency of IDS greatly. Additionally, the innovative machine learning methods rely on a large amount of labelled data that require considerable time and resources. Furthermore, checking that all Data is tracked before constructing the device may be essential. The exponential increase in statistical data advancement makes the traditional data processing scheme incredibly complex, imbibing much more effort and time. A big data technology is not perfect for unstructured data and imbalanced datasets. So more robust intelligent solutions are always in demand [1]. The challenge of classifying anomalies depending on network traffic patterns is critical for monitoring and recognizing advanced threat activities. Recently, PSO has gained extensive attention for its incredible outcomes in the composition of IDS [2].

Moreover, the assessment of variables has become an optimization challenge mainly in the practical implementation of PSO. IDS often interact with large volumes of data, potentially causing slow training and validation operation and poor detection levels. So, feature selection has become one of the essential themes in IDS research. An ML-based NIDS is a helpful solution for defending network services and infrastructure from unexpected and hidden threats. The IDS is the software or hardware configuration that primarily monitors, identifies, recognizes, and acknowledges specific intrusion and malicious behavior in a computer network. One of these kinds of alert data helps a manager to acknowledge and resolve the current system’s underlying issue [3].

**Figure 1:**
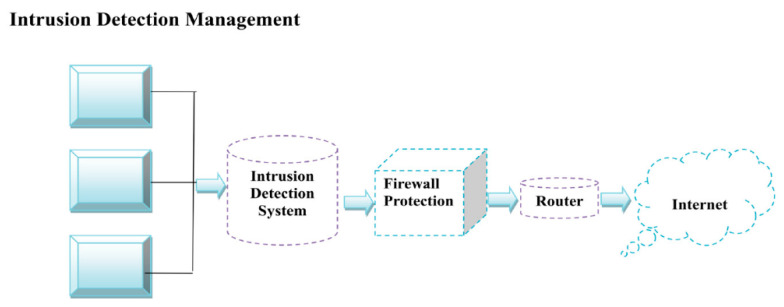
Working process of NIDS.

NIDS is a software and hardware device that recognizes suspicious attacks inside the network. Intrusion is categorized as anomaly-based or signature-based, depending on the recognition system. IDS development teams utilize a variety of techniques for intrusion detection and prevention. A few of these strategies are focused on machine learning methods. ML algorithms can forecast and identify attacks well before they turn into significant cyber threats. Binary categorization involves classifying objects into two groups [4]. Figure 1 shows the working of a NIDS. In IDS, a firewall and router occur between networks and devices.

On the other hand, multi-class categorization involves grouping occurrences into several categories. An ID is software or hardware-based application that monitors suspicious attacks or regulation violations upon a computer network or devices. As per the research [5], intrusion detection can be defined as follows: “The context of computer security, this same objective of which is to track the performance of the management system for both the occurrence of illegal purposes, i.e., behavior designed aimed to violate this same security plan regulating the confidentiality, integrity, and availability of service providers.” An intrusion detection system depends on various factors and characteristics. It can be divided into host-based intrusion detection systems (HIDS) and NIDS based on system type. A HIDS instance is a method for monitoring essential software files. Each HIDS helps detect an established intrusion by collecting and analyzing data (system log files and record-keeping of both the software) from a computer that hosts a platform, such as a user’s computer [6].

ML is a category of artificial intelligence (AI) that enables programs to improve overall accuracy at predicting events, despite being explicitly taught to do so. An intrusion detection system can utilize various methods to detect suspicious behavior in the dataset. Algorithms are implemented to recognize unknown threats. This scheme uses machine learning to generate a framework simulating activity and match changes in behavior with the solid framework. A computer algorithm has been assigned to perform specific tasks under machine learning. It has been concluded that the system has been improving since it started learning from its own experience. An ID system is a software that detects network intrusions by employing numerous machine learning methodologies [7].

The existing machine learning methods encounter several challenges, i.e., over-fitting and probing [8]. These methods are mainly trained on a single colossal dataset and require more time in data collection. Many existing ML methods do not support an automatic online learning process, requiring new training and consuming more computation power in the overall process [9]. This research develops an efficient hybrid network-based IDS model (HNIDS), which uses EGA-PSO and the improved random forest method. RF is a robust machine learning approach utilized in ML-based IDS. In the RF-based IDS detection, the model’s performance depends on two key parameters, minimal frequency occurrences and forest tree density. In practice, selecting these parameters is significantly challenging. We introduce an HNIDS that utilizes an EGA with PSO and IRF methods to overcome these challenges.

This essential contribution of this research is as follows:

1. This research develops an efficient model named “HNIDS,” which mainly deals with data imbalance and overfitting issues in the IDS dataset.
2. In the proposed HNIDS, we utilize EGA-PSO methods to improve the feature selection and detection accuracy.
3. To deal with the overfitting issues, this research utilizes the IRF method, which eliminates the less significant attributes, incorporates a list of decision trees across each iterative process, supervises the classifier’s performance, and prevents overfitting issues.
4. To evaluate the proposed HNIDS and existing ML methods, this research utilizes two benchmark IDS datasets, i.e., NSL-KDD and UNSW-NB15.
5. We have also calculated various key indicator parameters, i.e., precision, recall, F-measure, and accuracy for proposed HNIDS and existing ML methods.
6. In this research, we acquired 11 sub-attack variants over the DoS/DDoS threat class, 6 over the probe attacking class, and 7 with the users to root (U2R) threat class, including 15 sub-attack varieties including the remote to local (R2L) threat class.

The complete research article is organized as follows: Section 2 covers the literature review of the various existing IDS research based on ML methods. Section 3 covers the existing methods widely used in IDS. Section 4 covers the problem statement and proposed solution; Section 5 covers the dataset description, Section 6 covers simulation results and discussion, and the last section covers the conclusion and future direction of the research.

## 2. Literature Review

This section mainly reviews the various existing research in intrusion detection.

### 2.1. Review Based on ML and Deep Learning Methods

In the research [1], ML uses a statistical modeling strategy to learn previous data sequences and afterward anticipates one of the most likely outcomes utilizing novel statistics. Signature-based, anomaly-based, and hybrid-based detection are the three approaches that the IDS uses to detect intrusion. Signature-based detection identifies attack patterns by analyzing their signature [2]. In the research [3], to deal with this problem, anomaly-based identification, which relates consumer operations to predetermined statuses, is utilized to detect suspicious actions that could be incursions. Even without auto-updates, anomaly-based monitoring is helpful for unidentified or minimal assaults. However, this method usually has significant false-positive scores. In the research [4], a hybrid-based IDS employs several detection techniques to improve the performance of a specific methodology while gaining the benefits of multiple methods. Several studies have developed an automated approach for IDS to lower false-positive rates and provide efficient IDS. The research [5] proposed IDS predicated on deep AE and ML methodology. Essentially, the encoder portion of AE is used to enable the work in a non-symmetric manner, making it practical and efficient in computation time and cost. Two different non-symmetric deep AEs, only with hidden layers for each, were organized in a layered direction. A random forest algorithm was utilized for categorization. The KDD Cup’99 and NSL-KDD collections were utilized to investigate the classification contexts. The presented scheme proved its efficiency in terms of better accuracy rate and reduced time complexity over the existing DBN method. 

The research [6] also developed a similar concept of self-taught teaching methods predicated on sparse AE and a classifier. They completed experimental work on the developed framework, evaluating the NSL-KDD source data to authenticate their performance. The analysis indicates improvement in overall results compared to many DL and ML models. However, the proposed framework’s effectiveness in the R2L and the U2R category are not addressed. The research [7] considered various IDS predictions based on multiple nodes, sequence-to-sequence, structures, and filter spectrum AE, respectively. Such concepts were investigated for distinct benchmark datasets NSL-KDD, Kyoto, Honey pot, UNSW-NB15, IDS2017, and MAWILab vestiges. In research [8], the analysis indicates that the Seq2Seq model was formed utilizing multiple RNNs compared to other methods for overall classification accuracies and feature sets. The research [9] adopted the fundamentals of AE to introduce a multiphase model, incorporating the ID convolution operation and stacked layers. Within the preliminary unmonitored phase, two different AEs were trained and evaluated utilizing normal and attack streams to recreate the test results. Within the supervised phase, all new recreated specimens were utilized to construct a new enlarged dataset as feedback to a 1-dimensional CNN method. Table 1 reviews the various existing methods suggested by different researchers in intrusion detection.

**Table 1:** Review of existing work in the field of intrusion detection.

Reference	Methods/Techniques	Key Features	Challenges/Improvement
[10]	A hybrid method based on GA and ANN	Better precision and recall.	No real-time data set. Accuracy can be improved by adding two-way training. Analysis of variance (ANOVA) is missing.
[11]	Ensemble model based on meta-classification	Better precision and accuracy compared to other methods.	The training and testing process time is lengthy. The new IDS challenges were not covered.
[12]	Risk analysis of RPL and OFS	Capable of dealing with high-dimensional data.	Training requires a significant amount of time.
[13]	Deep learning in IDS	DNNs perform outstandingly in terms of better precision and recall.	Only limited datasets were used.
[14]	Deep-learning approach in NIDS	Reduce false alarms and training times.	ANOVA is not implemented. Only a few datasets were used.

### 2.2. Review Based on the Current State of the Art in IDS Research

The current developments and challenges concerning IDS in systems are examined about intrusion identification and control. The software and hardware modules that integrate multiple occurrences in systems for intrusion clues are “intrusion detection systems” [15]. Table 2 summarizes the current research in the field of IDS.

**Table 2:** The present state of the art in the field of intrusion detection research.

Key Method	References	Form of Benchmark Data	The Categories/Type of Intrusion in the IDS Dataset	Performance Measuring Criteria
Single technique-based recurrent neural network	[16]	Standard IDS data set	Probe, DoS, U2R and R2L	Precision, positive detection rate, false-positive rate.
	[17]	Standard IDS data set	SMTP, HTTP web, IAMP, TCP, ICMP, secure web, misapplication, IRC, Flow-Gen, ICMP, and DNS	Recall, F1-score, precision, AUC error rate, accuracy.
Machine learning methods	[18]	Standard IDS data set	Probe, DoS, U2R and R2L	True positive rate, accuracy, F1-score, precision, and recall.
	[19]	A real-time IDS data set	Probe, DoS, U2R and R2L	Accuracy, TPR, FP, TN, precision, TNR, recall.
	[20]	Standard IDS data set	Threats, malware, cyber threats	Accuracy and precision.
Evolutionary Machine Learning methods	[21]	Standard IDS data set	DoS, R2L, U2R and probe	Precision, recall true positive: false negative, false positive, true negative.
	[22]	Standard IDS data set	DoS, R2L, U2R and probe, SYN, threats, and DDoS	Precision, TPR, F1-score, accuracy.

### 2.3. Review Based on Symmetric Elements in IDS Research Using Machine Learning

The symmetry-adapted machine learning framework is a new AI system that involves information retrieval to reveal hidden correlations. It can retrieve and analyze information from ID schemes over the web, which also detects the presence of invisible and novel network threats to handle network security challenges and threats. Different machine learning algorithms, such as cluster analysis, association methods, and classification algorithms, may help extrapolate and find intrusion attempts for technology and information security concerns, such as malicious software, data leaks, malware, APTs, and data fusion.

#### 2.3.1. Dimensionality Reduction Challenges in IDS

The high-dimensional characteristics in computational problems contribute to lengthy classification procedures. On the other hand, low-dimensional characteristics can slow down these procedures [23]. Furthermore, the classification of internet traffic information with imbalanced class variances has presented significant limitations about the results that are attainable by most well-known classification models, which assume fairly balanced class distributions and equivalent miss-classification costs. The frequent occurrence and difficulties related to imbalanced classification models require additional scientific research. Earlier intrusion detection and prevention research does not address the issues related to NIDS classification due to imbalanced class patterns [24]. Table 3 represents the comparisons of the existing research based on dimensionality reduction in IDS.

**Table 3:** Comparison of existing research based on dimensionality reduction in IDS.

Reference	Method Used	Major Contribution	Challenges/Limitations
[23]	Principal component analysis	Able to manage large datasets, more efficient.	Not able to handle nonlinear problems.
[24]	Auto-encoders	It does not require any prior assumptions for the reduction.	Slower in speed.
[25]	Missing value ratio	Mainly finds out the missing values and NULL values.	Works on the specific data framework.
[26]	Low variance filter	It eliminates the low variance filter in specific dimensions.	Can work on limited data.
[27]	Factor analysis	It can analyze various data factors.	Slower.
[28]	Forward feature selection	It works in the forward direction.	Works on the specific data framework.
[29]	Uniform manifold approximation and projection (UMAP)	UMAP is crafted from a theoretical foundation predicated on Riemannian manifolds and algebraic configuration.	Can work on limited data.
[30]	Random forest	It constructs a dimension reduction tree based on the decision.	Can work on limited data.

#### 2.3.2. Concept Drift and Model Decay

Model drift and decay are principles that explain the workflow, all through which the efficiency of a model utilized for processing degrades on a genuinely innovative, new dataset, and the underlying principles show more about the difference in the value. Models should be retrained on novel inputs frequently [31]. Table 4 compares the current research for handling concept drift and model decay.

**Table 4:** Comparisons of current research for handling the concept of drift and model decay in IDS.

Reference	Research Scope	Major Contribution	Challenges/Limitations
[32]	Single classifier	It involves a single classification method.	Poor accuracy results.
[33]	Ensemble classifier for active state	It involves multiple classification active state methods.	More accurate and efficient.
[34]	Ensemble classifier for passive state	It involves multiple classification methods for passive state data.	More accurate.
[35]	Chunk-based data level	It involves actual attribute selection, employing continuously adaptive statistics analyses of input and probabilities of class labels.	It can work on specific data types.
[36]	Online learning based	It involves the online learning method.	Work on limited data.

## 3. Existing ML Methods

ML methods play a vital role in IDS research. This section covers the essential ML methods used in this research.

### 3.1. Logistic Regression (LR) Technique

LR is a procedure that involves designing the likelihood of precise results that are granted an input factor. A traditional LR model is built on binary class details, i.e., such as “yes/no, true/false.” Multiple linear regressions can model situations with more than two different outputs. LR is an effective analytical procedure for classification techniques, such as determining whether fresh samples belong in a specified group. LR is a valuable analytical method for areas of cyber security research that include classification problems, such as attack detection, IDS, etc. The LR technique is commonly used to analyze a sequence of object classes [35]. LR employs an operational function known as the “sigmoid function,” which is centered upon just a cost feature function. This function mainly helps in the mapping of specific probabilities with prediction values. A logistic regression equation for an input value y and weight values (β) predicts an output value x and input value y, as presented in Equation (1).

(1) $$x\;=\frac{e^{\left(a\beta 0+\;a\beta 1*y\right)}}{\left(1+{\;e}^{\left(a\beta 0+\;a\beta 1*y\right)}\right)}\;$$

### 3.2. Random Forest (RF) Technique

The RF classification is an ensemble technique that continuously uses bootstrapping, averaging, and bagging to train many decision trees. By employing distinct subsets of accessible characteristics, numerous independent decision trees can be constructed simultaneously on different segments of the training samples. Bootstrapping guarantees that any decision tree inside the random forest is distinct, lowering the RF variance. RF classification combines numerous tree decisions for the final judgment; as a result, the RF classifier has a strong generalization. The RF classifier aims to consistently outperform almost all other classifier techniques in terms of precision without difficulties of imbalanced datasets and overfitting [36]. A mean square error (MSE) for an RF can be defined as Equation (2).

(2) $$\mathrm{MSE}=\frac{1}{N}\overset{n}{\underset{k=0}{\sum }}\left(\begin{array}{c} n\\ k \end{array}\right)\left(\mathrm{Fi}-\mathrm{Yi}\right){\;b}^{2}$$

For Equation (2), N represents the number of distinct data points; Fi shows the outcome returned by model Yi and the precise value for point value is i.

### 3.3. Classification and Regression Tree Model (CART)

It is a forecasting framework for understanding how well the values of an outcome measure can be anticipated using information from other parameters. The CART total output is a predictive model in which each branch represents a divide in a response variable, so each ending node represents a result variable projection. In a CART model, a binary tree represents the items. To construct a CART structure, in the first step, we select input parameters and breakpoints on these parameters until an appropriate tree is no longer formed [37]. Equation (3) G (I) is the Gini index, ci is the class, and *cpi* is the class probability.

(3) $$G\;\left(I\right)=1-\overset{n}{\underset{i=1}{\sum }}\left(\mathrm{ci}+\mathrm{cpi}\right)$$

### 3.4. Linear Discriminant Analysis Technique (LDA)

LR is a prominent logistic classifier and uses well-used classification algorithms but struggles with multidimensional classification problems with very healthy categories. LDA, on the other side, manages these reasonably well. LDA, as with PCA, is used in text processing for dimension reduction and reduces the computational cost. Facial recognition methods utilize LDA well. LDA is still used in Fisher’s faces to retrieve specific information from numerous faces. LDA generates better results for binary images and also lowers the dimensionality issues. It represents variations, such as segregating data into various classes [38].

### 3.5. Support Vector Machine (SVM) Technique

The SVM is a supervised type of machine learning that solves classification performance issues with different classifiers. SVM features can classify novel content after giving training samples for each segment. The SVM classifier’s main objective is to discover a hyperplane inside an N-dimension internal space, where N represents the number of characteristics distinguished between the extracted features. The SVM method performs by linking statistics to a highly high vector space, enabling data points to be classified even when they are not quite linearly distinguishable usually. After performing a divider operation, the dataset is enhanced and can be easily partitioned and drawn as a feature space [39]. A square hinge loss value can be obtained by using (4), where y represents the actual value and $\overset{ˇ}{y}$ represents predicted value. A hinge loss is mainly used to determine any incorrect prediction in anomaly detection.

(4) $$L\left(y,\overset{ˇ}{y}\right)=\overset{n}{\underset{i=0}{\sum }}\;(\max\left(0,1-{\left(y,\overset{ˇ}{y}\right)}^{2})\right)$$

### 3.6. Naïve Bayes Technique (NB)

It is named Naïve since it automatically assumes that the particular characteristic’s existence is distinct from the event of specific other characteristics. If one supposes that the product’s color, structure, and flavor are used to identify things, in that case, a red, spherical, and juicy fruit is labeled as just an apple. As a result, each aspect helps to identify that it is an apple without relying on the others. An NB method focuses primarily on probability distribution and the hypothesis of further feature-complete independence. With every data set, the Naïve Bayes algorithm computes the likelihood function for distinct class and class sets [40]. It is termed Bayes because it is based on the Bayes’ Theory concept. Bayes’ theorem, often known as Bayes’ principle, is a mathematical formula for calculating the probabilities of a hypothesis with previous information. It is condition likelihood that determines this [41]. Bayes theorem can be defined as Equation (5). Here, P (A|B) shows the probability of “hypothesis A on the observed event B.”

(5) $$P\left(A|B\right)=\frac{P\left(A\right)P\;\left(B|A\right)}{P\left(B\right)}$$

## 4. Problem Statement and Proposed HNIDS Model

This section covers the problem statement identified based on the existing ML-based IDS research. It also covers the working of the proposed HNIDS model, algorithm, and the key strategy to resolve the identified issues.

### 4.1. Problem Statement

Based on the literature review in ML-based IDS, the following key issues are identified:

- Data imbalance: due to a limited training dataset in NSL-KDD, an ML-based IDS generates a higher false detection ratio and encounters data imbalance issues.
- Feature Selection: Another issue is related to the feature selection process.
- Performance: existing IDS are ineffective in dealing with new attack categories in networks due to their poor recognition rate and detection accuracy.
- Overfitting issues: overfitting is also a significant issue in IDS research.
- Issues related to the traditional random forest: in the RF-based IDS detection, the model’s performance depends on two key parameters, the minimal frequency occurrences and forest tree density, but in practice, selecting these parameters is significantly challenging.
- High computational overhead: The existing ML-based IDS encounters a higher computational time. In the existing ML-based IDS systems, it is essential to determine digital values for all the non-numerical fields in the NSL-KDD dataset, which helps in the normalization process [42].

### 4.2. Proposed HNIDS System

This research proposed an HNIDS that utilizes an EGA with PSO for feature extraction and IRF methods. We aim to determine the perfect features to improve the IDS performance, which includes significantly better precision and accuracy. The proposed HNIDS model will classify the attack category more precisely and efficiently.

### 4.3. Proposed HNIDS Working Steps

**Figure 2:**
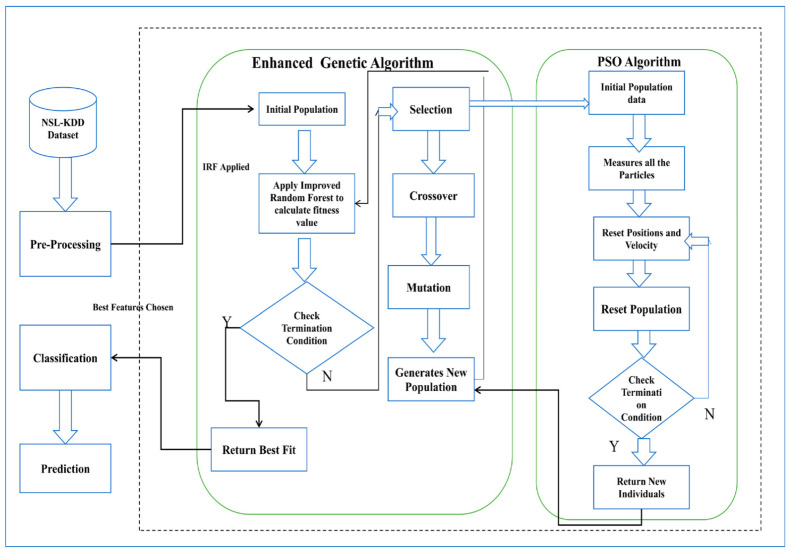
The working of the proposed hybrid IDS model.

A PSO method improves the vector in the proposed HNIDS. At the same time, the EGA is utilized to reconfigure the decision vectors that employ evolutionary operators. The proposed HNIDS is divided into the following stages: (1) data pre-processing, (2) By applying EGA-PSO, (3) By applying IRF, and (4) measures of the performance evaluation parameters. Figure 2 shows the architecture of the proposed system.

#### 4.3.1. Data Pre-Processing

An NSL-KDD database includes 38 numeric and three non-numeric values, including protocol type, service, and flag. Before initiating the feature selection process, the statistics must be normalized [43]. To accomplish this, we normalize the sample data attributes. This procedure has gained significance since all attributes may have varying data types. It mainly prevents significant numerical problems due to the variable parameters throughout the computation procedure [44]. We used min-max-based normalization within the proposed HNIDS method, which transforms a data value dv to dv’ in the limit (min_new_value to max_new_value), as described in (6).

(6) $$\mathrm{dv}=\frac{\mathrm{dv}-\mathrm{dv}\_\min}{\mathrm{dv}\_\max-\mathrm{dv}\_\min}\;\left(\max\_\mathrm{new}\_\mathrm{value}-\min\_\mathrm{new}\_\mathrm{value}\right)$$

In Equation (6), the range of the completely transformed attributes is denoted by min_new_value to max_new_value. In this research we used max_new_value 1 andmax_new_value to 0 andmin_new_value to 1. These transformed features are then utilized as input data for the feature selection technique.

#### 4.3.2. Apply EGA-PSO

Feature selection is one of the most crucial phases in IDS. The proposed model introduces a linear discriminant mutation approach based on statistical data analysis in EGA’s optimized mutation operator before applying a feature selection procedure. Then, the feature intervals are standardized using the min–max-based normalization process. The proposed HNIDS uses EGA to hunt for a new set of optimized features throughout the feature selection procedure by optimizing the EGA hyper-parameters and the revised selection operator [44].

Feature selection, a technique for obtaining a minimal group of adequate features, improves classification performance by evaluating the optimal sequence of elements from the basic feature set. It removes irrelevant features, reducing memory and computational overheads. It includes identifying a subcategory of features (SF) from the overall features (F) depending on a particular approximation algorithm. GA associated with PSO was utilized as a feature selection method to discover the best solution. John Holland was the first to use GA in 1975. However, they can be utilized to solve both search and optimization challenges. Particularly, GA comes under the umbrella of evolutionary algorithms (EAs). Across several complex real-world optimization project activities, EAs have conclusively proved to be the most extraordinary practical approach [45].

In 1995, Eberhart and Kennedy incorporated PSO for the first time. It is just a population-based optimal methodology focused on fish schooling and bird flocking behavior. The PSO method is the family member of swarm intelligence methodologies. Among the many PSO’s key benefits is that it becomes computationally efficient, owing to its fewer implementation requirements and cost. GA utilizes the legislation of genetic variation as its conceptual framework for integrating problem-solving across a population (P) of participants. Genes are a set of factors that characterize each participant. Genes are stringed together to form a chromosome. As a result, each real solution has a chromosome [46].

Existing is enhanced by adding a multi-objective function, which selects the best features and achieves improved fitness outcomes to explore the essential features and helps minimize dimensions, enhancing the true positive rate (TPR) and lowering the number of false positives (FPR). The proposed hybrid model also reveals a few symmetrical components, i.e., reduction, class imbalance, concept drift and model decay, and cross-validation to develop an effective IDS using ML [44].

The excluded participants from the selection process are forwarded to the PSO method for reformation. These excluded participants will link to upgrade their positions and velocities to ensure the optimal feasible outcome for the non-fit participants. Optimal PSO participants will be transmitted further into the current GA’s massive population. A fitness function across EGA and PSO is optimized, utilizing an optimized RF method to enhance the prediction performance.

The chromosomes Ch_i are encoded in a binary vector binary_i of length L in the HIDS, where i = (1,2,...,n). It utilized a binary encoding method to determine which feature was selected and not for the input data. The cluster of all chromosomes is mainly known as the “population.” The participant must have included critical attributes within the original population data (Ch_n, Slope, Ch_a, Thal).

Then, an EGA performs its assignment using critical operations of traditional GA, which are as follows: enhanced selection criteria, crossover process, enhanced mutation process, and higher fitness estimation. We believe that all the non-fit participants can have strong genes that can guide the selection module’s breakpoint to spaces in the solution space, where the most considerable enhancement can be discovered. As a result, the excluded chromosomes (non-fit participants) are forwarded to PSO for revolution, as GA tries to find healthy chromosomes rather than perfect genes. Based on the fitness value, all the healthiest participants are chosen depending on the validity of an optimization algorithm. It can be estimated across both PSO and EGA using the RF method with high precision to sustain the subsequent transmission [47,48].

Furthermore, RF prevents over-fitting, which is one of the primary issues in the IDS prediction process. As a result, the RF machine learning model is combined with EGA within the proposed HNIDS method to determine the best factors.

##### Enhanced Selection Process

In a GA process, the preliminary population is initialized at 50, and the highest counter value is 30 iterations. Afterward, we initiate the selection procedure, which is crucial for identifying the appropriate participants being acknowledged for one’s best fitness, from the current creation for re-production or even to be persisted in the subsequent stage [49]. The rejected participants are transferred to the PSO method for re-transformation. The above-rejected participants will form the PSO minority population, who can communicate to upgrade their positions and velocities to achieve the optimal outcome for the non-fit participants. The most acceptable PSO participants will be transmitted to the current GA population [50]. GA has used a modified fitness function by adding the features of the RF classification model. It helps to examine the scoring rate of each final solution.

A sequence must be decrypted into a binary sequence to determine the objective functions. An enhanced selection method is utilized with an initial size of 0.264. Even though the enhanced selection process is similar to the rank selection method regarding the sampling pressure, it is even more efficient in data processing and more adequate for parallel practical implementation. Individual fitness is determined through the following phases: (a) effectively transform all the data, feature set, and attributes in feature space, and (b) k-fold cross-validation process or instance validation with a high precision rating from the RF model. The sampling probability of each participant is determined by Equation (7), where the selection probability is ${\mathrm{Prob}}_{S}\;$, fitness value is ${\;\mathrm{Fit}}_{V}$, and n represents the nth chromosome value.

(7) $${\;\mathrm{Prob}}_{S}\left(n\right)=\begin{array}{c} \;\\ \; \end{array}\frac{{\mathrm{Fit}}_{V}\;\left(n\right)}{\sum_{k=0}^{n}{\mathrm{Fit}}_{V}\;\left(n\right)}{*\;\mathrm{Fit}}_{V}\;\left(n\right)$$

##### Enhanced Crossover Process

In this procedure, two different parent chromosomes are often utilized to create a new chromosome, mostly on-premise crossover operation probability, which is close to one in the experimental analysis. A string of formed chromosomes is preferable to its parent’s chromosome value. The stages of a complete crossover process are as follows:

- A pairing of two independent strings is selected with the guidance of its selection (re-production) operator.
- A cross-site is selected randomly and together with the width of the sequence.
- Apply to swap the string ranks and obtain the new sequence and positions.

##### Enhanced Mutation Operator Process

The strings initiate the mutation process after the crossover process is finished, which also seems to be a random transformation in a gene’s current value. A mutation involves changing one bit from zero to one or conversely. A mutation method improves an existing solution by significantly changing it. Mutation restricts the GA from becoming locked in a local solution. A mutation method is enhanced by incorporating a linear discriminant (LDM) method that relies on statistical data analysis. The hybrid HNIDS method that employs an EGA and PSO is addressed in Algorithm 1.
**Algorithm 1.** Proposed HNIDS method (EGA-with PSO)**Input:** attributes set, Random population (RP), the maximum number of generations (Max_g), Binary vector **Output:** Optimized individual generation RP(n)Step1: Apply RF method to determine the best fitness value for each participant   1.1 for each participant i to RP   1.2 do   1.3 determine the best fitness (i)       1.3.1 call random_forest ();   Step 2: Apply the enhanced selection process of GA   2.1 while (iteration_count< n)   2.2 call selection (i);   2.2.1 set New_selected_value = selection (i);   Step 3: Select best bit individuals using enhanced mutation and crossover (EGA)   3.1 Choose the best fit   3.1.1 if (New_selected_value == Best_fit), than   3.2 if (Crossover_generation) than   3.2.1 Choose two parents randomly (i_pa, i_pb)   3.2.2 Generates offspring parent (i_pc) = Crossover_generation (i_pa, i_pb);   3.3 else   3.4 Call enhanced mutation process   3.4.1 randomly select an independent value (i) from the parent set   3.4.2 Generates offspring parent (i_pc) = Crossover_generation (i_pa, i_pb);   3.5 else   Step 4: Calculate the best fit (fitness value) for each participant   4.1 if (New_selected_value == Best_fit)   4.1.1 Process Best_fit   4.2 else   4.2.1 Interchange the least fit participants with offspring parent (i_pc)   4.3 else   Step 5: Apply the PSO method to check the outcome best fir   5.1 New_Output_PSO = PSO_Rejected (offspring parent (i_pc))   5.2 End   Step 6: apply enhanced selection to generate a new population    6.1 if (New_Output_PSO == Best_fit) then   6.2 New_Output_PSO = New_Output_PSO + Current_PSO_Output   6.3 else   6.4 Call Enhanced_selection ();   6.5 End

#### 4.3.3. Apply IRF

In the proposed HNIDS method, IRF eliminates the less significant attributes, incorporates a list of decision trees across each iterative process, and supervises the performance of the classifier of the RF. The RF method is utilized for binary categorization in the proposed HNIDS method. RF creates a class with a mean predictive model after building numerous decision trees well in the training phase. A grid search method is used to groove all the hyper attributes of the RF method. We configure the numerous parameters for the RF method to implement the proposed HNIDS. In addition, the other parameters include 2 (min leaf size; 4 (min size for the split). The parameter value in the experiments includes max random trees: 1000, max depth: 10, Confidence: 0.5, belief:0.5 in the voting method, Gini impurity, pruning, and pre-pruning [51]. In addition, the other parameters include min leaf size: 2; min size for the split: 4. In the IRF method, a Gini impurity is determined by Equation (8), where *n* represents the number of classes used in the process, and RP represents the probability of selecting an element from class i data.

(8) $${\;\mathrm{Gini}}_{\mathrm{Impurity}}=\overset{n}{\underset{k=0}{\sum }}\mathrm{RP}\left(i_{\mathrm{pc}}\right)\left(1-\mathrm{RP}\left(i_{\mathrm{pc}}\right)\right)$$

Different decision trees operate together in an RF method as an ensemble form. An RF method means less computational cost and can build numerous minimal decision trees with limited features. We can integrate small decision tree structures into a single, robust candidate solution (large tree structure) by taking the average or possibly obtaining a significant percentage vote. The RF method is currently the most efficient training and learning method [52]. Algorithm 2 shows the function of the IRF method. In the proposed HNIDS method, the working of each of the methods is described in the following sub-sections.
**Algorithm 2.** Proposed HNIDS method (IRF working)**Input:** NSL dataset ds for training, v variable, n1 represents the total the nodes in a tree **Output:** Random Forest ensemble tree RF$\mathrm{Tree}\left(\begin{array}{c} k\\ 1 \end{array}\right)$
Step1: Construct an initial tree1.1 for each j to k repeat1.2 construct a sample set by using an initial IDS dataset (original) of size n1.3 start feeding the new bootstrapped dataset to a decision tree DFTreeStep2: Choose the best fit2.1 for each n1 to min node size, repeat2.2 start feeding the new bootstrapped dataset to a Random forest tree RFTree2.3 Select randomly variables v’, towards a variable set v2.4 Choose the fittest variable divided among all these variables v’2.5 divide a parent tree node into the new child nodes2.6 return ensemble tree RF$\mathrm{Tree}\left(\begin{array}{c} k\\ 1 \end{array}\right)$2.7 EndStep 3: Verify a constructed Random Forest ensemble tree RF$\mathrm{Tree}\left(\begin{array}{c} k\\ 1 \end{array}\right)$3.1 if (ensemble tree RF$\mathrm{Tree}\left(\begin{array}{c} k\\ 1 \end{array}\right)$ == Best_fit_tree BF$\mathrm{Tree}\left(\begin{array}{c} k\\ 1 \end{array})\right)$3.2 Proceed with the best fir ensemble treeStep 4: Determine the best class4.1 apply classification to find the best fit from BF$\mathrm{Tree}\left(\begin{array}{c} k\\ 1 \end{array}\right)$4.2 Calculate the number of votes4.3 if the number of votes is maximum4.4 return BF$\mathrm{Tree}\left(\begin{array}{c} k\\ 1 \end{array}\right)$4.5 End

#### 4.3.4. Measures of the Performance Evaluation Parameters

This researcher utilizes the following key parameters to measure the performance of the proposed HNIDS and existing ML methods [53].

- Accuracy: This evaluates the performance by the percent of the overall correctly classified instances. The percentage of correct forecasts is quickly measured by deducting them from the set of overall forecasts [54]. Equation (9) shows the formula for accuracy.
  (9) $$\mathrm{Accuracy}\;=\left[\frac{\left(\mathrm{TP}\;+\;\mathrm{TN}\right)}{\left(\mathrm{TP}\;+\;\mathrm{TN}\;+\;\mathrm{FP}\;+\;\mathrm{FN}\right)}\right]*100$$
- Precision: The precision calculates the proportion of accurate test scores penalized by the number of incorrect test scores [55]. Equation (10) shows the formula for precision.
  (10) $$\mathrm{Precision}\;=\left[\frac{\mathrm{TP}}{\left(\mathrm{TP}\;+\;\mathrm{FP}\right)}\right]*100$$
- Recall: The processing evaluates the actual number of classifiers penalized mainly through the number of records lost [56]. Equation (11) shows the formula for recall.
  (11) $$\mathrm{Recall}\;=\left[\frac{\mathrm{TP}}{\left(\mathrm{TP}\;+\;\mathrm{FN}\right)}\right]*100$$
- False alarm: The false alarm attempts to measure a false negative percentage of benign incidents as suspicious [57]. Equation (12) shows the formula for the false alarm.
  (12) $$\mathrm{False}\;\mathrm{Alarm}=\left[\frac{\mathrm{FP}}{\left(\mathrm{FP}\;+\;\mathrm{TN}\right)}\right]*100$$
- F-score: It is measured to limit the recall and precision correlation coefficient, providing an efficiency evaluation [58]. Equation (13) shows the formula for the F-score.
  (13) $$F-\mathrm{score}\;=\left[2\;*\;\left\{\frac{\left(\mathrm{Precision}\;•\;\mathrm{Recall}\right)}{\left(\mathrm{Precision}\;+\;\mathrm{Recall}\right)}\right\}\right]*100$$

where TP, TN, FP, and FN can be defined as follows:

◦ TP: It is known as “true positive”; the threat was predicted as just a significant attack.
◦ FP: It is known as “false positive”; actual data have been misclassified as just a significant attack.
◦ TN: It is known as “true negative”; metrics are classified correctly as a regular record.
◦ FN: It is known as “false negative“; standard metrics are correctly predicted as regular entries.

## 5. Data Set Description

We have chosen the NSL-KDD dataset [43] for this research because it is a benchmark dataset for network-based IDS and an improved form of the KDD-cup 99 dataset. Previous researchers have widely used the NSL-KDD data set [1,3,4,5], which motivates us to utilize the latest dataset.

There are 41 attributes and 1 class feature in the NSL-KDD data set. A total of 41 attributes play no role in attack detection, while others play a minor role. The NSL KDD dataset has the following advantages. No duplicate data/files exist in the NSL-KDD training dataset. So, a classification algorithm cannot generate any biased outcome. The classification algorithm can produce better reduction rates with no redundant log in the testing dataset. Combining multiple data from each complicated system element affects the percentage of the initial KDD data set records.

The training data comprise twenty-one attacks out of the same thirty-seven in the testing dataset. The existing dataset contains some extra attack fields. The following four attacks have been identified: probe U2R, R2L, and DoS. Table 5 shows the attack categories in the testing dataset [58,59].

An NSL-KDD dataset mainly contains KDD-Train^+^, KDD-Test^+^, and KDD-Test^−21^ datasets for training and testing. The dataset mainly includes two class categories based on classification type, i.e., binary and multi-class, as described in Table 6 and Table 7.

## 6. Simulation Results and Discussion

This section covers the experimental details, evaluation, and performance analysis of the proposed HNIDS and existing ML methods, i.e., SVM, RF, NB, LDA, and CART.

### 6.1. Simulation Setup and Details

Existing ML and proposed HNIDS methods are implemented using Python version 3.0 (https://www.python.org/download/releases/3.0/, accessed on 20 January 2022) in the Anaconda environment, on Windows 10 OS, with 8 GB RAM [60,61,62]. Various performance measuring parameters are calculated on the well-known IDS data sets NSL-KDD.

### 6.2. Results, Comparisons, and Validation

The proposed HNIDS model is compared to some well-known ML methods, including SVM, RF, LR, NB, LDA, and CART, using the NSL-KDD dataset. Some key measuring parameters (as discussed in Section 4) were calculated, i.e., TPR, TNR, FPR, FNR, accuracy, precision, F-score, false alarm, and recall rate.

In this research, an experimental analysis is performed in two stages. The first stage utilizes a BCC process. The BCC phase divides the network traffic into ‘normal-class’ and ‘abnormal-class.’

The second phase is based on an MCC. It utilizes a multi-class feature of network threats. An MCC is divided into the class categories of ‘normal,’ ‘DOS,’ ‘R2L’, ‘U2R’, and ‘probe.’

#### 6.2.1. Experiment 1: (Binary Class Classification)

An experimental first is performed to determine the BCC results for the NSL-KDD datasets (KDD-Train^+^, KDD-Test^−21^). Table 8 represents BCC’s outcomes for the proposed HNIDS and existing methods.

##### Observations and Discussion

Table 8 represents the experimental results of BCC on the KDD-Train^+^ dataset. The proposed HNIDS method archives accuracy of 98.979%, TPR99.658%, TNR 98.996%, precision 99.847%, FPR0.974%, FNR0.774%, recall 96.124%, false-alarm70.021%; F1-score 99.414%, which is higher than the existing ML methods. The random forest method recorded 97.498%, the second highest compared to other methods. Similarly, RF performs better in terms of TNR, precision, FPR, FNR, recall, false alarm, and F1-score than SVM, LR, NB, LDA, and CART ML methods.

**Figure 3:**
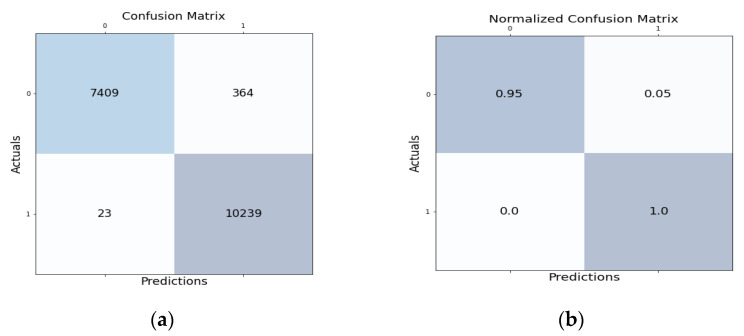
(**a**) Confusion matrix and (**b**) normalized confusion matrix for proposed HNIDS model for BCC.

Figure 3a shows the confusion matrix for the proposed HNIDS method for binary class classification, which shows TP as 7409, FP 23, FN 364, and TN 10239. Figure 3b shows that the normalized confusion matrix for the proposed HNIDS achieves TP of 0.95, FP 0.0, FN 0.05, and TN 1.0.

**Figure 4:**
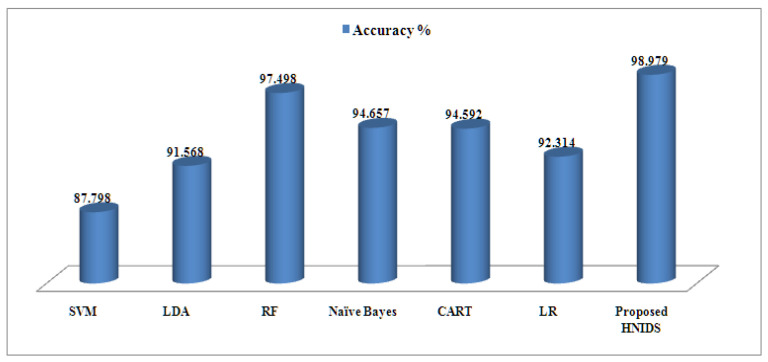
Accuracy % for proposed HNIDS model for BCC.

Figure 4 shows the accuracy graph for the proposed HNIDS and existing ML methods for BCC, which indicates the strength of the proposed method in terms of higher accuracy %.

#### 6.2.2. Experiment 2: (Multi-Class Classification)

Experiment 2 is performed to determine the MCC results for the NSL-KDD datasets (KDD-Train^+^, KDD-Test^−21^). Table 9 represents the outcomes of MCC for the proposed HNIDS and existing methods.

#### 6.2.3. Observations and Discussion

Table 9 represents the experimental results of MCC on the KDD-Train^+^ dataset. The proposed HNIDS method archives accuracy of 88.149%, TPR88.661%, TNR 87.996%, precision 82.867%, FPR 11.714%, FNR 10.414%, recall 90.447%, false-alarm 70.101%; F1-score 80.478%, which is higher than the existing ML methods. The Naive Bayes method recorded 86.771%, the second highest compared to other methods. In addition, SVM recorded 87.148% TPR, 85.998% TNR by random forest, 78.719% precision by CART, 15.623% FPR by LR, 11.121% FNR by RF, 88.936% recall by RF, 71.457% false alarm rate, 80.584% F1-score, which are the best outcomes after the proposed HNIDS.

**Figure 5:**
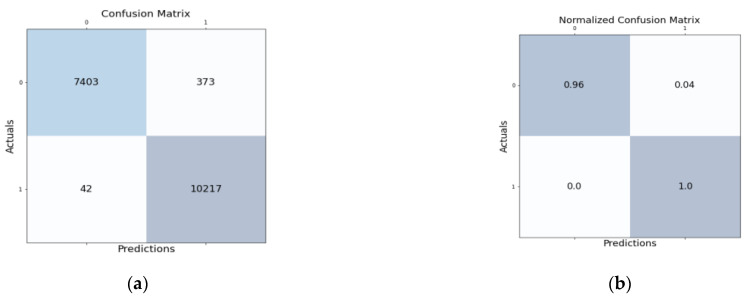
(**a**) Confusion matrix and (**b**) normalized confusion matrix for the proposed HNIDS model for MCC.

Figure 5a shows the confusion matrix for the proposed HNIDS method for multi-class classification, which shows TP as 7403, FP 42, FN 373, TN 10217, and Figure 5b shows the normalized confusion matrix of the proposed HNIDS. Proposed HNIDS achieves TP of 0.96, FP 0.0, FN 0.04, and TN 1.0.

**Figure 6:**
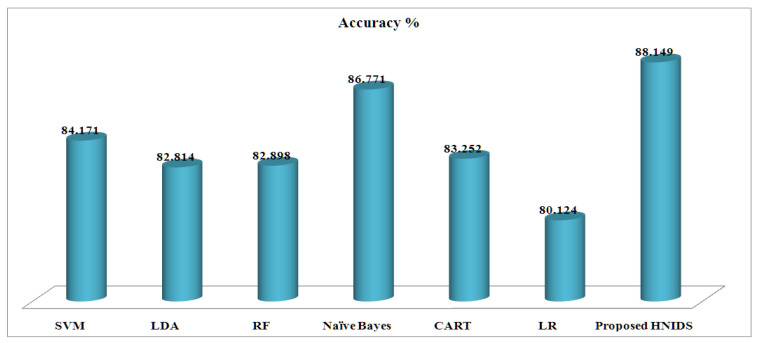
Accuracy % for proposed HNIDS model for MCC.

Figure 6 shows the accuracy graph for the proposed HNIDS and existing ML methods for MCC, which indicates the strength of the proposed method in terms of higher accuracy % over the existing ML methods.

## 7. Conclusions and Future Works

Developing an efficient ID using the ML method has earned enough attention for cyber security in previous research. The security database often contains multiple features, which are redundant or even meaningless. Developing a new feature selection method is now necessary, decreasing the computational overhead and expenses and enhancing the classification performance. This research mainly deals with data imbalance issues in IDS research. This research developed an HNIDS based on EGA-PSO and IRF methods. Based on the literature review, we found that an NSL-KDD dataset is widely used for network-based IDS, so we decided to utilize the same dataset in this research. An experimental analysis was performed, and various performance measuring parameters were calculated, i.e., accuracy, TPR, TNR, precision, FPR, FNR, recall, false alarm, and F1-score. The proposed HNIDS achieved better results than the other ML methods, i.e., SVM, RF, LR, NB, LDA, and CART.

In current IDS research, accuracy, TPR, precision, and FPR are the four most widely used performance measurement parameters. In future research, we will explore more comparison parameters, i.e., CPU utilization, latency, and detection, to evaluate the performance of the proposed HNIDS on a real-time IDS dataset. We will also use parallel processing to analyze large IDS data sets, reduce detection time, and increase operational efficiency.

## Figures and Tables

**Table 5:** Description of the NSL-KDD dataset.

Attack Classes in NSL KDD Dataset	Types of Attacks
Probe class	IP-sweep, Satan, Port-sweep, N-map, Saint, and M-scan
DoS class	Land, Back, Tear-drop, Pod, Neptune, Smurf, Worm, Mail-bomb, Process-table, Apache-2, and Upstorm.
R2L class	Ftp_write, Guess_password, Phf, Imap, Warez-master, Multi-hop, Xlock, Snmpguess, Xsnoop, HTTP-tunnel, Snmpget-attack, Named, and Send-mail.
U2R class	Buffer_overflow, Xterm, Rootkit, Loadmodule, Sqlattack, Perl, and Ps

**Table 6:** Binary class classification (NSL-KDD) dataset.

Data Set Type	Normal Record	Abnormal Record	Total Records
KDD-Test^−21^ dataset	2125	9698	11,850
KDD-Train^+^ dataset	67,343	58,630	1,25,973
KDD-Test^+^ dataset	22,544	9711	12,833

**Table 7:** Multi-class classification (NSL-KDD) dataset.

Data Set Type	Normal-Class	DoS-Class	Probe-Class	R2-L Class	U2-R Class	Total Records
KDD-Test^−21^ dataset	2125	4344	2421	2885	67	11,850
KDD-Train^+^ dataset	67,343	45,927	11,656	9,95	52	1,25,973
KDD-Test^+^ dataset	9711	7460	2421	2885	67	22,544

**Table 8:** Experimental result of BCC on KDD-Train^+^ dataset.

Classification Method	Accuracy %	TPR %	TNR %	Precision %	FPR %	FNR %	Recall %	False Alarm %	F1-Score %
SVM	87.798	96.748	95.656	97.847	3.147	2.968	91.414	91.242	96.665
LDA	91.568	93.447	91.774	96.778	2.987	3.145	90.565	78.127	96.321
RF	97.498	97.658	95.998	97.847	2.747	3.541	94.665	91.334	97.478
Naïve Bayes	94.657	96.114	92.045	95.624	2.147	2.747	93.554	79.542	95.078
CART	94.592	92.474	94.141	96.547	4.145	2.878	92.778	81.632	95.621
LR	92.314	90.852	90.784	91.263	3.564	2.145	90.112	80.471	91.256
Proposed HNIDS	98.979	99.658	98.996	99.847	0.974	0.774	96.124	70.021	99.414

**Table 9:** Experimental results of MCC on KDD-Train^+^ dataset.

Classification Method	Accuracy %	TPR %	TNR %	Precision %	FPR %	FNR %	Recall %	False Alarm %	F1-Score %
SVM	84.171	87.148	85.656	78.417	17.477	12.878	83.659	81.442	86.005
LDA	82.814	85.701	81.774	74.814	18.657	13.557	82.447	79.477	86.201
RF	82.898	82.601	85.998	77.457	16.047	11.121	88.936	71.445	83.008
Naïve Bayes	86.771	84.457	82.045	75.478	17.107	14.877	85.223	77.502	85.968
CART	83.252	85.441	84.141	78.719	18.547	13.978	86.445	76.694	83.771
LR	80.124	83.278	80.117	75.961	15.623	11.451	80.978	71.457	80.584
ProposedHNIDS	88.149	88.661	87.996	82.867	11.714	10.414	90.447	70.101	83.478

## Data Availability

The dataset is available at: https://www.unb.ca/cic/datasets/nsl.html, accessed on 20 January 2022.

## Abbreviations

This result utilizes the following abbreviations:

Acronyms	Definitions
ANN	Artificial neural network
ANOVA	Analysis of variance
CART	Classification and regression tree model
DoS	Denial of services
DDoS	Distributed denial of services
EGA	Enhanced genetic algorithm
FPR	False positive rate
HIDS	Host-based intrusion detection system
HNIDS	Hybrid network-based intrusion detection system
IDS	Intrusion detection system
IRF	Improved random forest
LDM	Linear discriminant method
LDA	Linear discriminant analysis technique
LR	Logistic regression
NSL-KDD	Network security laboratory-knowledge discovery in databases
ML	Machine learning
NB	Naïve Bayes
PSO	Particle swarm optimization
R2L	Remote to local
SVM	Support vector machines
TPR	True positive rate
